# A study to evaluate the impact of COVID-19 on Lifestyle of Medical students

**DOI:** 10.12669/pjms.38.7.6031

**Published:** 2022

**Authors:** Sania Mumtaz Tahir, Nazish Imran, Imran Ijaz Haider, Ali Burhan Mustafa, Aqeeb ur Rehman, Muhammad Waqar Azeem, Afzal Javed

**Affiliations:** 1 Sania Mumtaz Tahir, MBBS. Post Graduate Resident, Academic Department of Psychiatry and Behavioral Sciences, King Edward Medical University/Mayo Hospital, Lahore, Pakistan; 2 Nazish Imran, MBBS; FRCPsych (London); MRCPsych (London); MHPE. Professor, Department of Child and Family Psychiatry, King Edward Medical University/Mayo Hospital, Lahore, Pakistan. Member World Psychiatric Association Presidential Working Group on Medical Students; 3 Imran Ijaz Haider, (FRCPsych, MRCPsych, DPM (UK), Professor of Psychiatry & Behavioural Sciences, Fatima Memorial Hospital, Lahore, Pakistan; 4 Ali Burhan Mustafa. FCPS (Psych), DCP (Ireland), Associate Professor of Psychiatry & Behavioural Sciences, Sheikh Zayed Medical College/Hospital, Rahim Yar Khan, Pakistan; 5 Aqeeb-ur Rehman. 4th Year Medical Student, King Edward Medical University, Lahore, Pakistan; 6 Muhammad Waqar Azeem, MD, DFAACAP, DFAPA. Chair and Professor, Department of Psychiatry, Sidra Medicine, Weill Cornell Medicine-Qatar, Doha, Qatar. Chair, World Psychiatric Association Presidential Working Group on Medical Students; 7 Afzal Javed, FRCPsych, MRCPsych. Chairman Pakistan Psychiatric Research Centre, Fountain House, Lahore, Pakistan. Honorary Professor Institute of Applied Health Research, University of Birmingham, UK. President, World Psychiatry Association

**Keywords:** COVID-19, Lockdown, Diet, Sleep, Physical activity, Screen time

## Abstract

**Background:**

Stressful situations are often linked with poor health and lifestyle behaviors (e.g., unhealthy diet, limited physical activity, poor sleep quality etc.). Subsequent to the nationwide lockdown in Pakistan due to COVID-19 pandemic, medical students experienced substantial lifestyle changes along with academic stresses. The goal of this study was to measure the impact that the COVID-19 lockdown had on lifestyle like sleep, physical activity and nutrition, substance abuse, dealing with finances, spirituality and family life, with respect to a sample of Pakistani medical students.

**Methods:**

This cross- sectional online study involving 1100 medical students (68.7% females) from five medical colleges in Pakistan, used self-administered questionnaire to evaluate the impact of pandemic related restrictions on lifestyle of medical students in Pakistan from August- September 2020. Parameters such as physical activity, sleep, nutrition, smoking and substance abuse, family life, finances, internet use and spirituality were studied.

**Results:**

Fifty-nine percent of medical students reported a significant effect of lockdown on their physical activity. Only 5.8% people clearly followed or adhered to a routine during the pandemic. About 54.6% students have found that physical activity and exercise significantly reduced their anxiety. More than half of respondents reported increase in screen time, weight gain and poor sleep with 3.1% of students stating an increased use of sleeping pills to fall asleep Another 18.3% students had an increased worry regarding COVID19 based on the information they received on internet. No significant increase in substance uses and alcohol use or family conflicts among students post pandemic was noted.

**Conclusion:**

Our findings underscore that COVID-19 pandemic has led to undesired changes in health and lifestyle habits of medical students, which may, to some extent, be responsible for higher negative impact. Hence it is important for medical colleges to start awareness campaigns to tackle challenges for medical students physical and emotional well-being.

## INTRODUCTION

Coronavirus pandemic forced most parts of the world to go in strict lockdown as a preventive measure against the spread of the disease.^1^ This resulted in closing of all non-essential establishments including universities and colleges. Pakistan Government announced the closure of all educational institutions on 13th March 2020 in the first wave of the COVID-19 pandemic. Educational institutes closed again as a part of national lockdown during the second wave in Pakistan on November 25th, 2020. This resulted in rapid shift towards e-learning platforms to continue providing education, including medical education.

Early studies have shown a great deal of negative impact of lockdown measures on mental health of medical students.^2^,^3^ Stress and poor mental health is directly associated with poor sleep, unhealthy eating and increase in substance abuse in people, especially susceptible among them are the college students.^4^ About 67% of Italian medical students had decreased physical activity due to prolonged sitting and sedentary behaviors post-lockdown.5 16% of the students were sleeping for less than seven hours.^5^ Increased life stress has also been linked to unhealthy eating.^6^,^7^ Therefore, we expected a shift towards unhealthy eating habits during lockdown among the students. Young adults have reported a significant increase in substance abuse during pandemic with 38% of users reporting severe drug abuse.^8^ In a study conducted in United States on college students, it was found 26.7% students reported increase in alcohol use since the start of pandemic.^4^ It is important to study the changes in lifestyle of medical students due to multiple reasons. Firstly, it is important for students to improve their personal health, and secondly, those who adopt healthy lifestyle can be better promoters of healthy behavior for their patients as future physicians.

Information on the real impact of the lockdown and confinement on medical students’ lifestyle is lacking, and although studies regarding the effect of the lockdown on mental health and Knowledge and attitudes are starting to appear,^9^,^10^ scant research has been published focusing on lifestyle (nutrition, physical activity, screen time and sleep etc.) during pandemic. This information would allow medical institutions to develop better public health promotion strategies and recommendations for lifestyle modifications. The goal of this study was to measure the impact that the lockdown period due to COVID-19 had on lifestyle, of a sample of Pakistani medical students.

## METHODS

The study protocol was approved by the Institutional Review Board of King Edward Medical University (No: 719/RC/KEMU). Participants completed an anonymous online survey through google forms web survey platform commencing in August 2020 for six weeks. The convenience sample (sample size n=1100) was recruited by communicating the link to the survey form through the official channels of involved institutions, contacting the student representatives of classes as well as social media (WhatsApp group of medical students in researchers’ institutions). Prior to participation, the aim of survey was explained along with assurance of response confidentiality and compliance with ethical standards. No reward was offered to respondents for taking part in the survey. Informed consent was taken and participants were given the freedom to withdraw from the study at any time without having to give reasons. Inclusion criteria was being enrolled in any year in participating medical colleges. Students who failed to provide consent or who did not complete the questionnaire were excluded.

The survey was co-developed with input from World Psychiatric Association (WPA), Presidential action group on medical students’ members as well as medical students’ representatives and literature available on the topic. Participants provided information about socio-demographic characteristics (age, gender, place of residence, college year, number of people in the household during the confinement) etc. The influence of COVID-19 Pandemic on sleep, physical activity and nutrition, substance abuse, dealing with finances, spirituality and family life using Likert scales (Questionnaire used available as supplemental file).

All statistical analyses were done using SPSS (SPSS Version 20.0, SPSS Inc., Chicago, IL). Descriptive statistics were calculated for whole sample as shown in Table-I. Impact on different areas is represented as numbers and percentages.

**Table I:** Demographic characteristics of Respondents. (N=11,00).

Characteristics	No (%)
Age: Mean (S.D)	23.14 (6.31)
***Gender***	
Women	756 (68.7)
Men	344 (31.3)
***Marital Status***	
Single	1084 (98.6)
Married	12 (1.1)
Divorced/widowed	4 (.4)
***Year in Medical College***	
First year	259 (23.6)
Second year	187 (17)
Third year	266 (24.2)
Fourth year	182 (16.5)
Final year	206 (18.8)
***Place of residency during the pandemic***	
City	948 (86.2)
Rural/village	152 (13.9)
***No. of people residing in the house during Pandemic***	
1 (I live alone)	13 (1.2)
2	21 (1.9)
3	56 (5.1)
4	157 (14.3)
5 or more	853 (77.5)

**Table II:** Impact of COVID-19 Pandemic on Lifestyle & Family life of Medical Students. PHYSICAL ACTIVITY

Statements	Not at all (n) %	A little bit (n) %	Moderately (n) %	Much (n) %	Very much (n) %
Do you consider that exercise is important during this pandemic?	168 (15.2%)	366 (33.2%)	266 (24%)	204 (18.5%)	94 (8.5%)
Does exercise help you in the prevention of anxiety?	53 (4.8%)	164 (14.9%)	282 (22.9%)	339 (30.8%)	262 (23.8%)
Have you increased the frequency and intensity of your physical workout during this epidemic and lockdown?	397 (36%)	285 (25.9%)	214 (19.4%)	131 (11.9%)	73 (6.6%)
How much has your physical activity been affected by this pandemic of COVID-19?	178 (16%)	263 (23.9%)	240 (21.8%)	219 (19.9%)	200 (18%)
***NUTRITION***					
Did you notice the need to eat larger amounts of food or eat more often?	I eat much less than I used to. (n) %	I eat bit less than I used to (n) %	Neither more nor less (n) %	I eat a bit more than I used to (n) %	I eat much more than I used to (n) %
	112 (10.2%)	155 (14.1%)	385 (35%)	337 (30.6%)	111 (10.1%)
Please mark the answer that best represents your eating habits during this period.	I eat in a healthier way (n) %		My eating habits and preferences have not changed (n) %	I eat in an unhealthier way (n) %	
	262 (32.9%)		379 (34.5%)	359 (32.6%)	
***SLEEP***					
The quality of my sleep has changed recently. It is:	Much worse (n) %	A little bit worse (n) %	The same (neither worse nor better) (n) %	A little better (n) %	Much better (n) %
	201 (18.3%)	359 (32.6%)	330 (30%)	135 (12.3%)	75 (6.8%)
	Almost never (n) %	Rarely (n) %	Some times (n) %	Often (n) %	Almost always (n) %
I tend to stay up late and sleep for many hours during the day.	102 (9.3%)	165 (15%)	274 (24.9%)	240 (21.8%)	319 (29%)
I take sleeping pills to help me sleep at night.	933 (84.8%)	72 (6.5%)	61 (5.5%)	19 (1.7%)	15 (1.4%)
I am having dreams in which I feel trapped, over the last 3 weeks.	547 (49.7%)	227 (20.6%)	192 (17.5%)	86 (7.8%)	48 (4.4%)
***INTERNET USE***					
Statements	Not at all (n) %	A little more (n) %	Moderately more (n) %	Much more (n) %	Too much (n) %
The information and use of the internet worry me about the issue regarding the COVID-19:	300 (27.2%)	408 (37%)	190 (17.2%)	110 (10%)	92 (8.3%)
Generally, most of the internet sources regarding information about COVID-19 are misinforming/misleading:	226 (20.5%)	438 (39.8%)	233 (21%)	117 (10.6%)	86 (7.8%)
Due to the epidemic conditions, the internet takes up more of my time than usual:	95 (8.6%)	225 (20.4%)	169 (15.3%)	247 (22.4%)	364 (33%)
	Yes (n) %		No (n) %		
Have you acquired internet-related habits that you did not have before (for example: created a facebook account, engaging in or gambling)?	443 (40.3%)		657 (59.7%)		
***SMOKING, ALCOHOL AND SUBSTANCES USE***					
Smoking before the Pandemic	I didn’t smoke (n) %		I was smoking (n) %		
	1063 (96.6%)	37 (3.4%)
Use of Illicit substances (e.g. alcohol, hashish, cocaine etc) use before the Pandemic	I did not use it (n) %		Occasionally and rather rarely (n) %	Often (n) %	
	1,071 (97.4%)	20 (1.8%)	9 (0.8%)
***FAMILY***					
	Much less	Less	Same	More	Much more
Do you feel the need to communicate with other members of your family during this period?	59 (5.4%)	128 (11.6%)	395 (35.9%)	359 (32.6%)	159 (14.5%)
.Are there any conflicts with the rest of your family members during this period?	285 (25.9%)	227 (20.6%)	310 (28.2%)	202 (18.4%)	76 (6.9%)
Do you want to receive emotional support from other members of your family during this period?	113 (10.3%)	127 (11.5%)	336 (30.5%)	334 (30.4%)	190 (17.3%)
Has the overall quality of relationships with the other members of your family changed compared to before the COVID – 19?	Much worse	Worse	It has not changed	A little bit better	Much better
	17 (1.5%)	127 (11.5%)	563 (51.2%)	262 (23.8%)	131 (11.9%)
***FINANCES***					
How are your finances as a result of the outbreak?	Much more difficult than before	Somehow more difficult	Same as always	Somehow easier	Much easier than before
	141 (12.8%)	379 (34.5%)	510 (46.4%)	56 (5.1%)	14 (1.3%)

## RESULTS

Eleven hundred students (68.7% female) from various academic years in five different medical colleges participated in the online survey. The rest of demographic details are given in Table-I. About 7% reported that they are *generally able* to maintain a basic daily routine, i.e. waking up in the morning, regular meals and sleeping hours and activities (either alone or as family). Another 40.2% reported that they were able to follow it *somehow but not always*. While 28.3% people were *not at all* able to follow a routine, only 5.8% people *clearly followed or adhered to* a routine during the pandemic

**Fig.1:**
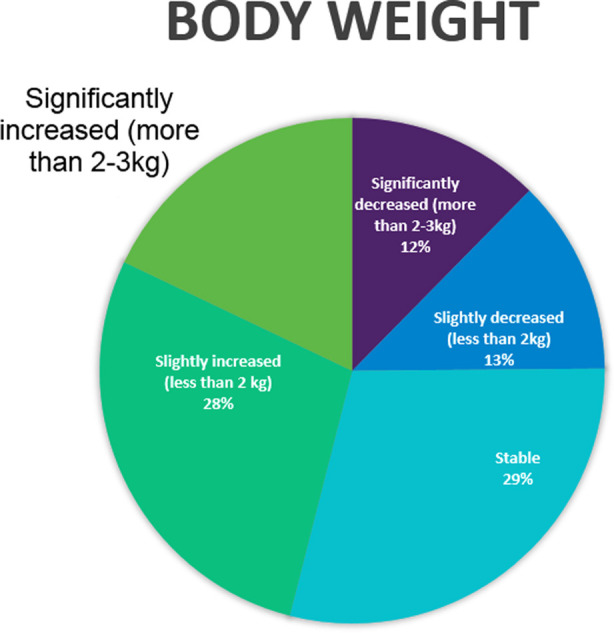
Changes in body weight reported by medical students during the COVID-19 Pandemic.

**Fig.2:**
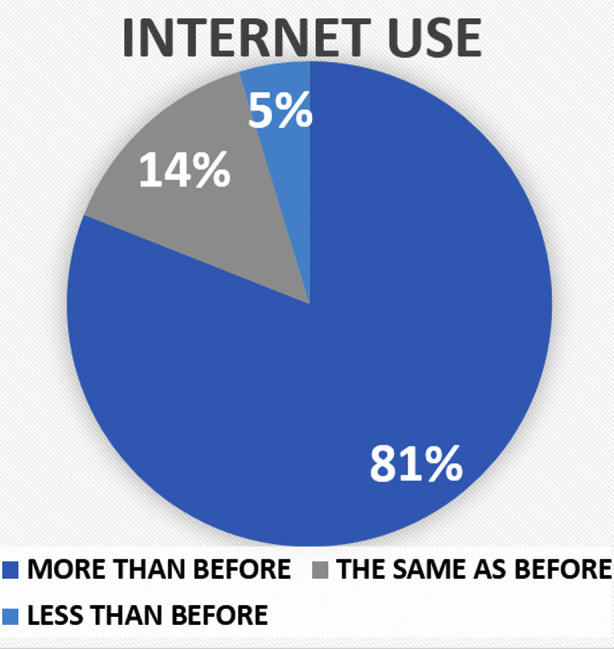
Changes in internet use reported by medical students during the COVID-19 Pandemic.

The details of changes in lifestyle in medical student’s lives during the pandemic, including changes in physical activity, sleep, nutrition, family life, finances, internet use, smoking and substance use are enlisted in Table-II. Changes in weight, and internet use reported by medical students during the pandemic are described in Fig.1 & 2. Almost 60% reported that their physical activity has been affected by the pandemic to some extent and no significant change was noted in smoking or alcohol and substance use. More than half of respondents reported increase screen time, weight gain and poor sleep with 3.1% of students stating increase use of sleeping pills to fall asleep.

Among the respondents, 32.4% reported an increase in their religious/spiritual inquiries over past 2-3 weeks, 40.6% respondents said their religious/spiritual inquiries increased *a little bit*, while 26.9% respondent noticed *no increase* in their religious/spiritual inquiries.

## DISCUSSION

To our knowledge, this is the first study conducted in Pakistan at a larger scale (sample =1100) to study the impact of Corona Virus Pandemic on various lifestyle behaviors in medical students (such as physical activity, sleep, nutrition and body weight, family life, substance use and smoking and spirituality).

### Physical Activity

Our study showed various aspects of physical activity perceptions and behaviors in our participants ranging from the perception of importance of exercise during the pandemic to the effect of pandemic on their personal physical activity. Among 1100 students who responded to the survey, 51% believed exercise was important during the pandemic. Studies have found an inverse relationship between anxiety and physical activity in view of the start of Covid-19 pandemic.^11^ In our study, 54.6% students have found physical activity and exercise significantly reduced their anxiety and 22.9% students report a moderate reduction in anxiety due to exercise which is consistent with the above-mentioned inverse relationship between exercise and anxiety.

As we predicted early on, there had been a significant effect of the pandemic on the physical activity of the students with 59.7% reporting that their physical activity has been affected by the pandemic to some extent. This is consistent with the findings in the study conducted on the Italian medical college students where 67% students reported a decrease in physical activity with increase sitting time.^6^ Barkley et al., 2020 showed that physical activity varied among the students differently based on their pre-pandemic activity level.12 As our study doesn’t have the pre-pandemic activity data so it is not possible to comment on it.

### Nutrition

Maintaining a healthy diet is of prime importance during the pandemic as building a good immunity is the second line of defense against COVID-19, after preventive measures like wearing a mask and social distancing. But several factors could contribute to inability to maintain a healthy diet. Firstly, due to lockdown access to fruits and vegetables may be limited. Secondly, since lockdown negatively effects the mood and depression, anxiety and stress may lead to ‘stress eating’ or ‘emotional eating.^8^,^13^

Our study found that 40.7% students noticed that they have been eating more often than they used to. In addition to that, 49.8% students reported an increase in body weight. These findings are consistent with Di Renzo 2020, where 48.6% population had a perceived gain in weight, with 40.3% reporting slight increase and 8.3% people reporting greatly increased weight.^13^

With regard to food choices, the distribution was more or less equal with 32.6% people eating unhealthier than before and 32.9% consuming food that was healthier than before and 34.5% reported no changes in food choices. Di Renzo in 2020 however showed that only 15% of the people in the Italian population were able to opt for fruits and vegetables.^13^ This could be because of stricter lockdown measure and limited access to food in Italy as compared to Pakistan.

### Sleep

According to current recommendations, an adult should sleep at least 7 hours a day to lead a healthy life. We studied the changes in quality of sleep, sleep duration, sleeping patterns, use of any somnolent drug and the nature of dreams in our study. Around 50.9% students reported that their quality of sleep was much worse than before. The effect of pandemic on quality of sleep has been previously widely documented with significant worsening seen in Chinese population.^10^ A change in sleeping habit was reported by 50.8% students with sleeping for several hours during the day and staying up late at night. This finding is significant because maintaining a good sleep hygiene and circadian rhythm has a positive effect of one’s mental health and consequently, one’s immune system.

There has been a significant increase in students (69%) reporting dreams, with the theme of being trapped. There has been an increase in such dreams globally with people dreaming of being trapped in boxes or restricted freedom since the start of pandemic which are due to the psychological effect of lockdown measures and stay at home order.^14^

### Internet Use

With the start of pandemic, there has been a rapid spread of relevant and sometimes irrelevant information which has led to increasing the fear surrounding this disease. Dubbed as the ‘infodemic’,^15^ this rapid spread of information may increase the anxiety associated with the spread of disease. However, it has been found that fear of COVID-19 is inversely proportional to health literacy in the medical students with students with more knowledge having less fear.^16^ Our study found that only 18.3% students had an increased worry regarding COVID-19 based on the information they received on internet.

In addition, 18.4% students felt like the information shared online was misleading. This misinformation can be damaging to the disease control efforts of social distancing by feeding into the online conspiracy theories. Since COVID-19 is a communicable disease, people led by the misinformation are not only putting themselves in danger but also putting others in danger as well.^15^

Since the shifting of educational activities online and stay at home orders, 55.4% students reported a significant increase in their internet use. Fernandes B, et al. 2020 reports an increase in use of internet and social media among the youth with an increase in tendency towards internet addiction and gaming addiction.^17^ Same is the pattern seen in social media use where the participants in our study reported increase in use in 81% individuals, with 40.3% students engaging in activities they previously didn’t engage in before such as making an account on a certain platforms or gambling. While some social media use can be beneficial and has supported medical students,^18^ it is widely known fact that social media has negative impact on mental health and concentration.

### Smoking, alcohol, and substance use

To understand the trend of use of cigarettes, alcohol and other substances during lockdown, we first asked the trend of use before the lockdown in which only 3.4% reported to be smoker and 2.6% were using alcohol or other drugs. Siddiqi K, et al, 2020 has seen a bidirectional change in smoking patterns in smokers in a longitudinal study comparing pre and post pandemic results.^19^ Our study shows similar finding in students with 1.9% smoking more than before and 2.6% smoking less than before. This bidirectional change was also consistent in use of alcohol and other substance use. (2.1% more than before, 1.7% less than before) These changes are somewhat different than expected as previous studies have shown a significant increase in substance abuse and alcohol use among students post pandemic.^20^ One of the reason for difference between these could be the difference in social and cultural setup between the two study populations as well as the availability of these substances in Pakistan, with all these drugs and alcohol being largely illegal and travel and trade restriction can hinder the ease of access.

### Family

With closure of all colleges and hostels, all students had to move back home and quarantine with their respective families. Living with a family during the pandemic has been shown to be a protective factor against anxiety related to COVID-19 among students.^21^

Since the start of quarantine, there has been a general idea of increase in family conflicts due to stressful life events, economic conditions and spending too much time together. However, Evans S, 2020 finds in his qualitative study on Australian families during lockdown that each family has a varied experience with some families suffering from mental health issues and family conflicts to some reporting improved relationship with family.^22^ Our finding correlates with this varied experience among the respondents.

### Spirituality and Psychological reflections

As a part of coping in natural disasters it has been seen that spirituality and practicing religion reduces the psychological suffering and gives an alternate coping mechanism.^23^ Our study shows 32.4% students reported an increase in religious/spiritual inquiries in past 2-3 weeks and 40.6% students reporting a little increase in spiritual/religious inquiries.

Study has various clinical implications. In view of COVID-19, these lifestyle patterns are quite concerning as it shows a decline in physical activity, lack of sleep and increase in weight and smoking which are all risk factors for a severe COVID infection. Moreover, adoption of such lifestyle behaviors by medical students is likely to affect their education and training. As future physicians, they have the responsibility of promoting healthy lifestyle in others. There is a need to promote and support healthy lifestyle behaviors. This can be done via public awareness campaigns, lectures, counselling and feedbacks sessions.

### Limitations

This study was conducted online via forms circulated through social media platforms. Therefore, one limitation of the study is that it is a cross sectional analysis, with no pre-pandemic data and no follow up available to see the changes in patterns before and after this period to see and track the trends. Another limitation of this study, is the use of a questionnaire rated with a Likert scale instead of clinical scales to measure physical activity and other parameters like Pittsburg Sleep Quality Index (PQSI) etc.

## CONCLUSION

In the wake of changing world situation, identifying the changes in behaviors is important as they have far reaching effects and implications. Our study identified changes in such behaviors in medical students in Pakistan. They reported to have an increased tendency towards sedentary lifestyle, with approximately 60% students having decreased physical activity than before. In addition, 50.9% students had poor sleep and altered sleep patterns. Changes in eating pattern led to an increase in weight of up to 50% students. There has been an increase in smoking and other substances of abuse as well. Overall, such patterns raise concerns as they are not only poor prognostic factors in COVID infection but also can affect medical students’ life and education in the long run. This study highlights the need to improve education, provide support and raise awareness regarding lifestyle behavior and their changing patterns so that students may make healthier choices.

### Authors’ Contributions:

**NI, MWA, AJ** conceived the idea of this study.

**NI, SMT, IIH, ABM, AR and ZK** collected and analyzed data.

**NI, SMT & IIH** wrote the first draft of the manuscript.

**NI, MWA & AJ** were responsible for the overall supervision of this project.

All authors reviewed and edited the manuscript and approved the final version of this article.
